# Unveiling the therapeutic effects of traditional Chinese patent medicines: A network meta-analysis on chronic atrophic gastritis

**DOI:** 10.1097/MD.0000000000041690

**Published:** 2025-03-07

**Authors:** Baoping Ren, Meiqi Zhong, Chang Yu, Meng Xiong, Shunhua Zhou, Qing Gao, Xiaojuan Wang, Qinghua Peng, Meiyan Zeng, Mengzhou Xie, Houpan Song

**Affiliations:** a Hunan Provincial Key Laboratory of Traditional Chinese Medicine Diagnostics, Hunan University of Chinese Medicine, Changsha, Hunan Province, China; b College of Traditional Chinese Medicine, Hunan University of Chinese Medicine, Changsha, Hunan Province, China; c Key Laboratory of TCM Heart and Lung Syndrome Differentiation & Medicated Diet and Dietotherapy, Hunan University of Chinese Medicine, Changsha, Hunan Province, China.

**Keywords:** chronic atrophic gastritis, evidence strengths, network meta-analysis, randomized controlled trials, traditional Chinese patent medicine

## Abstract

**Background:**

The study aimed to conduct a network meta-analysis of randomized controlled trials (RCTs) to examine the effectiveness and safety of traditional Chinese patent medicine (TCPM), either used alone or combined with conventional treatment (CT) of chemical drugs, for treating chronic atrophic gastritis (CAG).

**Methods:**

We searched the literature from database creation to December 2023; our primary endpoint was clinical response rate. Secondary outcome measures were the inhibition rate of *Helicobacter pylori* and clinical symptom score, including pain and noisy scores for the stomach, score for belch and acid reflux, score for the bitter taste and the dry throat, and safety according to total adverse events. Data analyzed using RevMan 5.3, R (v4.0.2), and Stata SE 15.0. Mean differences (MDs) for continuous outcomes and odds ratios (ORs) for dichotomous outcomes and drug ranking by *P*-score. Network meta-analysis presented as forest plot and league table. Results reported as MDs/ORs with 95% confidence interval (CI).

**Results:**

Our analysis comprised 49 RCTs with 5218 patients. Compared to using CT alone, all TCPMs + CT performed better in improving clinical response rate. The Weifuchun tablet (WFCT) inhibition rate (OR = 9.05; 95% CI = 1.89–43.34; *P*-score = .92) was relatively high, but only 1 RCT supported this result. WFCT + CT (OR = 2.94; 95% CI = 1.97–4.39; *P*-score = .57), Qizhiweitong granule (QZWTG) + CT (OR = 2.91; 95% CI = 1.07–7.89; *P*-score = .55), and Moluodan (MLD) + CT (OR = 2.44; 95% CI = 1.37–4.36; *P*-score = .43) had certain advantages in inhibiting *H pylori* compared to using CT alone. Compared with CT, Weisu tablet (WST) + CT (OR = 0.21; 95% CI = 0.05–0.89; *P*-score = .24) and Xiangshayangwei pill (XSYWP) + CT (OR = 0.41; 95% CI = 0.18–0.92; *P*-score = .44) were drugs that were less likely to cause adverse reactions.

**Conclusion:**

Our results demonstrate that, compared to using CT alone, TCPMs combined with CT can improve the clinical response rate in the treatment of CAG. Additionally, some TCPMs combined with CT can enhance the *H pylori* inhibition rate. WST + CT and XSYWP + CT can even reduce the occurrence of adverse reactions.

## 1. Introduction

Chronic atrophic gastritis (CAG) is a common chronic inflammatory condition of the mucosa of the stomach and is often associated with the infection of *Helicobacter pylori*.^[1]^ CAG can cause gastrointestinal symptoms, including abdominal pain, indigestion, loss of appetite, and increased risk of gastric cancer (GC).^[2]^ Therefore, CAG can be managed by identifying *H pylori* infection as the primary pathogenic factor. Additionally, recognizing its significant role in preventing GC is equally important.^[3]^ The primary pharmacological interventions for treating CAG include the clinical administration of antibiotics, proton pump inhibitors, antacids, and gastric mucosal protective drugs.^[4]^ These medications can effectively ameliorate mucosal inflammation and the associated symptoms, such as abdominal pain. However, the traditional recommended plan for inhibiting *H pylori* infection has certain limitations in its effectiveness. Accordingly, traditional Chinese patent medicine (TCPM) has emerged as a promising alternative to treating CAG with *H pylori*; it has the functions and advantages of improving the *H pylori* inhibition rate, reducing adverse reactions and antibiotic usage, and improving the pathological changes of gastric mucosa.^[5]^

Clinical studies have shown that TCPMs can alleviate CAG symptoms, slow disease progression, and reduce side effects.^[6,7]^ Moluodan (MLD), a condensed pill of TCPM, improves CAG by inhibiting inflammatory responses and regulating cell proliferation and apoptosis.^[8]^ Weifuchun tablet (WFCT) can mitigate CAG and impede precancerous lesion development in GC by regulating various signaling pathways, including HMGB1/NF-kB, RUNX3/TGF-beta/Smad, Hedgehog, and Wnt.^[9]^ Administrating Weisu tablet (WST) as a therapeutic intervention for CAG induced by *H pylori* infection can ameliorate symptoms, mitigate inflammatory responses, enhance *H pylori*-negative conversion rate, and augment treatment effectiveness.^[10]^ Qizhiweitong granule (QZWTG), a traditional Chinese herbal prescription originating from the Sinisan decoction in Shang Han Za Bing Lun, has significant clinical effectiveness in CAG treatment within the Chinese population.^[11]^ The effectiveness of Xiangshayangwei pill (XSYWP) in treating patients with CAG has been proven. It can alleviate clinical symptoms, reduce inflammatory reactions in the body, optimize various immune function indicators, effectively improve the nutritional status of patients, and reduce the incidence of adverse reactions during treatment.^[12]^ Table S1, Supplemental Digital Content (http://links.lww.com/MD/O440) lists the involved ingredients of TCPMs. However, the effectiveness of diverse TCPM treatments is controversial; TCPM, as an exclusive medical system in China, has a rich history of treating gastrointestinal disorders through several therapeutic approaches and classic prescriptions. Nevertheless, owing to the variations in TCPM treatments and the absence of standardized research methodologies, the efficacy and safety of TCPM treatments for CAG remain uncertain.^[13]^

Network meta-analysis enables the amalgamation of evidence from various studies, thereby facilitating indirect comparisons between treatments that have not undergone direct head-to-head trials.^[14]^ This methodology can synthesize data from multiple randomized controlled trials (RCTs) on different treatments and estimate their comparative effects.^[14,15]^ Consequently, network meta-analysis provides a more comprehensive understanding of the efficacy and safety of various treatments. This study aimed to conduct a network meta-analysis on RCTs focusing on TCPM interventions for CAG. The purpose was to evaluate the efficacy and safety of various TCPM therapies for CAG.

## 2. Methods

There was strict adherence to the preferred reporting items for systematic reviews and meta-analyses guidelines for conducting systematic reviews and network meta-analyses.^[16]^ The registration number is CRD42024484844. Since this study did not involve human or animal testing or case reports or series of cases, the Ethics Committee and Institutional Review Board did not have to approve it.

### 2.1. Search strategy

To select appropriate research for incorporation into our network meta-analysis, we conducted a comprehensive search across multiple databases, including China National Knowledge Infrastructure (CNKI), PubMed, WanFang, VIP, and Embase. The search encompassed publications released from database creation and December 2023. To ascertain further relevant research, we comprehensively searched the references of published systematic reviews and trial summaries. The scope of our investigation was restricted to scholarly works exclusively published in Chinese. We searched these databases using the search strategy with the following keywords: (“CAG”) AND Intervention = (WFCT or WST or MLD or QZWTG or XSYWP or conventional treatment (CT) of chemical drugs).

### 2.2. Study selection

The studies included in this network meta-analysis met the following inclusion criteria: be RCTs; patients diagnosed with CAG (All sexes, races, and ages were included); have an observation group treated with a TCPM or a TCPM + CT and a control group treated with CT. A clear preparation manufacturer and approval number were required for the TCPMs.

Conversely, this network meta-analysis excluded the studies that met any of the following inclusion criteria: the observation group received decoctions, herbal extracts, or synthetic plant-based drugs; the observation group included acupuncture, moxibustion, and massage, rather than TCPMs; prescription composition with unknown dose of intervention drugs and unmatched treatment method.

### 2.3. Data extraction

Two researchers independently screened the literature using the Cochrane Collaboration Systematic Evaluators manual (version 5.1.0). NoteExpress was used to review the titles and abstracts of the literature. The full literature text was obtained and read after removing irrelevant and duplicate literature. The third researcher discussed or judged differences in the included literature after cross-checking. The information extracted from the data included demographic characteristics, diagnostic criteria, randomized method, allocation plan, treatment and control measures, drug dosage, drug composition, trial period, and clinical efficacy. Changes after intervention were equal to the endpoint value minus the baseline value for each intervention.

### 2.4. Quality assessment and risk of bias

The Cochrane risk of bias tool was employed to evaluate the study level assessment. According to the Software Review Manager 5.3, 2 review authors autonomously screened search results, extracted data from the included studies, and evaluated their risk of bias. Seven items were included in the risk of bias evaluation, and each item was rated either as high risk, low risk, or unclear. The third reviewer resolved the discrepancies if a consensus could not be reached.

### 2.5. Data synthesis and analysis

With the statistical package “net meta” in R (version 4.0.2), a network meta-analysis was performed using a frequentist approach to include direct and indirect comparisons. Meanwhile, Stata SE 15.0 software was used to create relevant diagrams.^[17]^ The network meta-analysis results can provide a more accurate estimate of the effect than pairwise meta-analyses and can be used to rank drugs based on their efficacy for a given outcome, guiding clinical decisions.^[18,19]^

First, the network evidence figure was presented to illustrate the comparison between the interventions; a diagram of nodes representing drugs and connections shows the available direct comparisons between pairs of drugs. The size of a node was determined by the number of patients randomized to a specific drug, whereas the size of a connection was determined by the number of trials that compared 2 drugs. Furthermore, to examine publication bias and other effects of small studies, Stata SE 15.0 was employed to create comparison-adjusted funnel plots. A scatter plot of the effect size versus the precision, measured by the inverse of the standard error. If the effect estimate line is symmetrical, there is no publication bias or small effect. The consistency model is used if no difference exists.^[20]^ To determine whether the data could be pooled, the *I*^2^ statistic was used to quantify heterogeneity. In cases where *I*^2^ was over 50%, the random effects model was used for the meta-analysis after analyzing the underlying source and eliminating clinically significant heterogeneity. Nevertheless, for a more conservative estimate of TCPMs efficacy, data were pooled using a random effects model.

Mean differences (MDs) were used for continuous outcomes, and odds ratios (ORs) were used for dichotomous outcomes. The network meta-analysis was performed as a forest plot and a league table. The results were presented as MDs or ORs, with a 95% confidence interval (CI). When assessing dichotomous outcomes, treatments associated with an increased OR were favored, indicating a high effective rate of TCPMs. Treatment with a more significant mean reduction from baseline was preferred for the continuous outcomes of pain and noisy scores for the stomach, belch and acid reflux score, and bitter taste and dry throat score. Positive values indicated an increase with treatment, while negative values indicated a reduction; the lower the negative value, the greater the favor. *P*-score mean ranks were used to rank the treatments for each outcome; the higher the score, the greater the likelihood of the drug being ranked as the best, but *P*-score magnitude should also be considered.^[21]^ As the mean value of the *P*-score is always .5, drugs clustering around this value are likely to have similar efficacy. Instead of relying on rankings alone, it is necessary to consider the ORs and 95% CIs for each comparison when interpreting the results. The netsplit was used when evaluating the inconsistency of specific cyclic methods.^[22]^

To assess the confidence in the direct and indirect treatment estimates of the network for our primary endpoint of improving clinical effectiveness in CAG, we employed the confidence in network meta-analysis (CINeMA) framework, which the Cochrane Collaboration endorses. A sensitivity analysis was carried out to address potential heterogeneity and assess the resilience of the findings. Within this framework, we utilized the risk of bias from missing evidence in the network meta-analysis tool to evaluate reported bias (Fig. S1, Supplemental Digital Content, http://links.lww.com/MD/O440).

## 3. Results

### 3.1. Study search and characteristics

**Figure 1.**
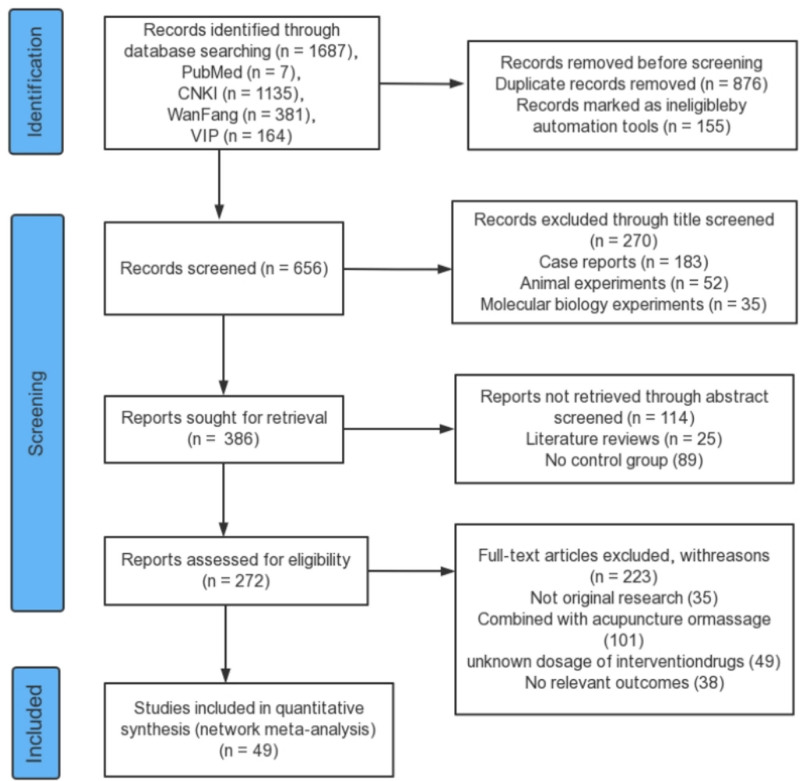
Flow diagram of preferred reporting items for systematic reviews and meta-analyses (PRISMA) 2020.

Our prespecified retrieval strategy revealed 1687 relevant articles, A total of 1031 articles were excluded through a duplicate check and automated tools, and 384 articles were excluded based on their incompatible titles and abstracts with the prespecified intervention. Eventually, 272 articles were downloaded that met the criteria after preliminary screening; we excluded 223 articles from our review that did not meet our inclusion criteria, leaving 49 articles (Fig. 1). Of the 49 RCTs included articles, there were 4 RCTs about WFCT, 19 RCTs about WFCT + CT, 11 RCTs about MLD + CT, 4 RCTs about XSYWP, 5 RCTs about QZWTG + CT, 5 RCTs about XSYWP + CT, 2 RCTs about WST, and 2 RCTs about WST + CT. There were 49 Chinese articles involving 9 interventions. The experimental group was treated with TCPM alone or combined with CT, and the control group was treated with CT; all articles were 2-armed. In total, 5218 subjects were enrolled, 2605 in the experimental group and 2613 in the control group; the subjects were aged 17 to 80. Data from all studies were comparable at baseline (Table S2, Supplemental Digital Content, http://links.lww.com/MD/O440).

### 3.2. Risk of bias

Of the 49 included RCTs, 6 RCTs presented a high risk of bias, of which 4 RCTs were evaluated in a nonstandard randomization process, and 2 studies were considered to present a high risk of bias in incomplete outcome data. Unfortunately, none of the studies reported allocation concealment and blinding, potentially compromising their reliability. Each item represented a certain proportion of the total (Fig. S2, Supplemental Digital Content, http://links.lww.com/MD/O440).

### 3.3. Primary outcomes

**Figure 2.**
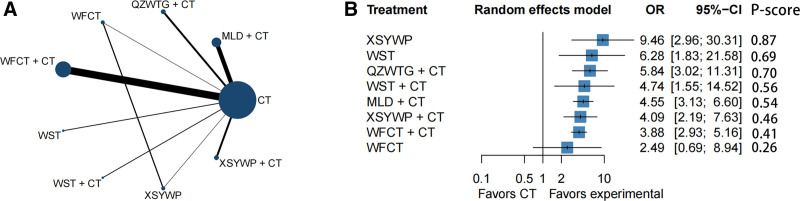
(A) Network plot for clinical response rate in chronic atrophic gastritis (CAG). The circle (node) size is proportional to the number of study participants assigned to receive each intervention. The line width (connection size) corresponds to the number of studies comparing the individual interventions. (B) Forest plot for clinical response rate in CAG. The *P*-score is the probability of each intervention being ranked best in the network. CT = conventional treatment of chemical drugs, MLD = Moluodan, QZWTG = Qizhiweitong granule, WFCT = Weifuchun tablet, WST = Weisu tablet, XSYWP = Xiangshayangwei pill.

**Figure 3.**
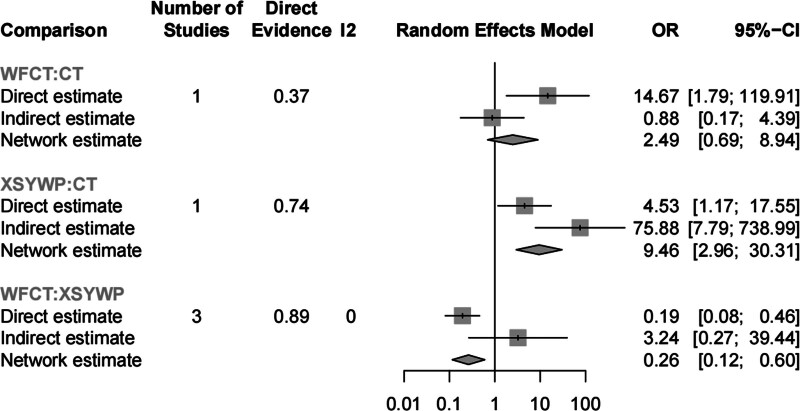
Netsplit diagram. CT = conventional treatment of chemical drugs, WFCT = Weifuchun tablet, XSYWP = Xiangshayangwei pill.

**Figure 4.**
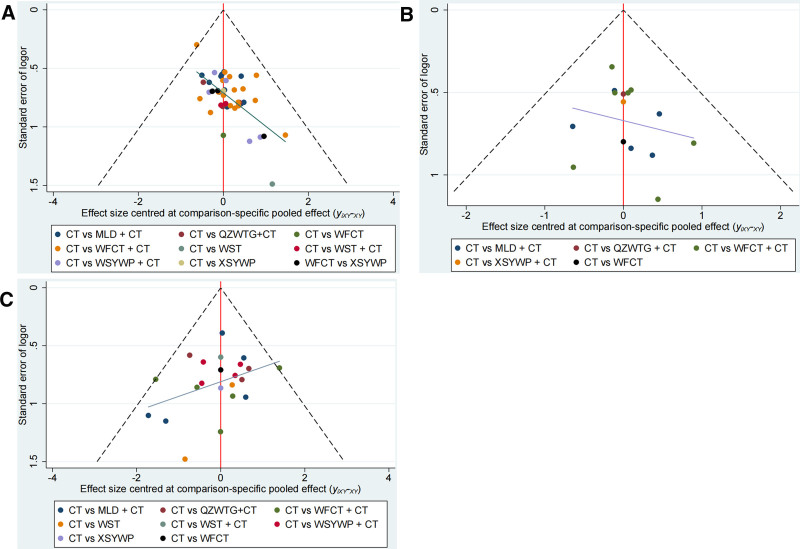
Adjusted funnel plot for (A) clinical response rate, (B) Inhibition rate of *H pylori*, and (C) adverse events. CT = conventional treatment of chemical drugs, MLD = Moluodan, QZWTG = Qizhiweitong granule, WFCT = Weifuchun tablet, WST = Weisu tablet, XSYWP = Xiangshayangwei pill.

The present study analyzed 49 RCTs^[23–71]^ involving 5218 patients to report on a specific endpoint. Figure 2A presents a network plot, while low heterogeneity (*I*^2^ = 0%) was observed when combining the data. There was insignificant inconsistency when evaluating the inconsistency of specific cyclic methods, with *I*^2^ > 0 and *P* ≥ .05 (Fig. 3). Furthermore, the adjusted funnel plot in Figure 4A does not reveal publication bias or minor study effects. XSYWP ranked first for the clinical response rate (OR = 9.46; 95% CI = 2.96–30.31; *P*-score = .87), indicating that the probability of XSYWP being the most efficacious drug was 87%. QZWTG + CT (OR = 5.84; 95% CI = 3.02–11.31; *P*-score = .70) and WST (OR = 6.28; 95% CI = 1.83–21.58; *P*-score = .69) were considered to have some degree of efficacy and were identified as the second and third relatively effective drugs, respectively. Compared to CT alone, all TCPMs + CT showed better performance in improving the clinical response rate (Fig. 2B). Except for WFCT, there were statistically significant differences compared to CT. Direct and indirect comparisons indicated that XSYWP might have an advantage over WFCT. There was no statistically significant difference in indirect comparisons between other TCPMs (Fig. S3A, Supplemental Digital Content, http://links.lww.com/MD/O440). When using the CINeMA framework to evaluate the reliability of outcomes of this endpoint, most direct and indirect comparisons within the network were found to have relatively low or moderate confidence levels (Table S3, Supplemental Digital Content, http://links.lww.com/MD/O440). Sensitivity analysis showed that if any point estimate after deleting a study falls within the 95% CI of the total effect size, the study is considered to have a small impact on the combined effect size, indicating that the result is relatively robust (Fig. S7, Supplemental Digital Content, http://links.lww.com/MD/O440).

### 3.4. Secondary outcomes

**Figure 5.**
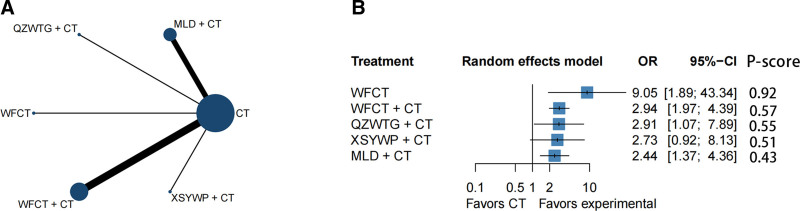
(A) Network plot for inhibition rate of *H pylori* in chronic atrophic gastritis (CAG). The circle (node) size is proportional to the number of study participants assigned to receive each intervention. The line width (connection size) corresponds to the number of studies comparing the individual interventions. (B) Forest plot for inhibition rate of *H pylori* in CAG. The *P*-score is the probability of each intervention being ranked best in the network. CT = conventional treatment of chemical drugs, MLD = Moluodan, QZWTG = Qizhiweitong granule, WFCT = Weifuchun tablet, WST = Weisu tablet, XSYWP = Xiangshayangwei pill.

In our study, 2 secondary outcome indicators were included, namely the *H pylori* inhibition rate and the symptom score. The *H pylori* inhibition rate was evaluated through a network plot of 15 RCTs,^[23,25,27,30,32–34,37,47,48,50–52,62,65]^ involving 1542 patients (Fig. 5A). The adjusted funnel plot (Fig. 4B) suggests that there may be no significant publication bias. The pooled data showed relatively low heterogeneity (*I*^2^ = 0%). The WFCT inhibition rate (OR = 9.05; 95% CI = 1.89–43.34; *P*-score = .92) was relatively high, but this is based on a single small-scale trial. The treatment ranked second for *H pylori* inhibition was WFCT + CT (OR = 2.94; 95% CI = 1.97–4.39; *P*-score = .57). QZWTG + CT (OR = 2.91; 95% CI = 1.07–7.89; *P*-score = .55) was potentially considered as the third relatively effective treatment. MLD + CT ranked fourth (OR = 2.44; 95% CI = 1.37–4.36; *P*-score = .43). All TCPMs + CT, with the exception of XSYWP + CT, showed some degree of superiority over CT, and compared with using CT alone, it has statistical differences (Fig. 5B). Figure S3B, Supplemental Digital Content, http://links.lww.com/MD/O440 indicates that there may not be a significant difference between the TCPMs in direct and indirect comparison.

A few studies have been conducted to compare symptom scores of CTPMs. However, the limited number of studies included in these analyses precluded the assessment of publication bias. Given the few interventions, the present study was restricted to a direct comparison meta-analysis. Based on 4 studies^[29,31,33,41]^ encompassing 578 patients, it was found that WFCT + CT was more effective than CT in alleviating bitter taste and dry throat (MD = –0.77; 95% CI = –0.98 to –0.57) (Figure S4A, Supplemental Digital Content, http://links.lww.com/MD/O440). Two studies^[29,31]^ comprising a total of 240 patients have demonstrated the superiority of WFCT + CT score over CT alone in assessing gastric noise (MD = –4.75; 95% CI = –5.25 to –4.25) (Fig. S4B, Supplemental Digital Content, http://links.lww.com/MD/O440). In 4 studies^[29,31,33,41]^ encompassing 578 patients, the effectiveness of WFCT + CT in reducing belching and acid reflux was superior to CT (MD = –6.53; 95% CI = –10.97 to –2.09) (Fig. S4C, Supplemental Digital Content, http://links.lww.com/MD/O440). Figure S5A, Supplemental Digital Content (http://links.lww.com/MD/O440), shows the network plot. The pooled data indicated a low level of heterogeneity (*I*^2^ = 7%). The results of 6 studies,^[26,29,31,33,41,62]^ which included 720 patients, demonstrated that the combination of whole food plant-based diet and cognitive therapy WFCT + CT was more effective than cognitive therapy alone in reducing the pain score associated with stomach pain (MD = –0.68; 95% CI = –1.00 to –0.34) (Fig. S5B, Supplemental Digital Content, http://links.lww.com/MD/O440). However, the difference between XSYYW and CT was insignificant.

### 3.5. Adverse events

Adverse events were reported in 22 RCTs^[23,25,32,37,40,41,44,46,48,49,52,53,55,58,59,61,63,66–70]^ recruiting 2130 patients. Data pooling showed low heterogeneity (*I*^2^ = 23%). Furthermore, the adjusted funnel plot (Fig. 4C) does not reveal publication bias or minor study effects. The likelihood of WST being associated with adverse events was the lowest (OR = 0.12; 95% CI = 0.02–0.57; *P*-score = .11), which means that the probability of WST being the least likely drug to cause adverse reactions was 89%. However, only 2 RCTs reported adverse reactions to WST, which may result in relatively large errors in the results due to the small sample size. Compared with CT, WST + CT ranked second (OR = 0.21; 95% CI = 0.05–0.89; *P*-score = .24) and XSYWP + CT ranked third (OR = 0.41; 95% CI = 0.18–0.92; *P*-score = .44) among the drugs least likely to cause adverse reactions. The differences between the 3 drugs mentioned above and CT were statistically significant. QZWTG + CT (OR = 0.86; 95% CI = 0.35–2.13; *P*-score = .78) was a drug that seemed more likely to be associated with adverse events, although its CI exceeds 1, indicating that the difference is not significant compared to CT (Fig. S6, Supplemental Digital Content, http://links.lww.com/MD/O440). After direct and indirect comparisons, WST was less likely to cause adverse reactions than MLD + CT and QZWG + CT, with only these 2 comparisons showing statistically significant differences (Fig. S3C, Supplemental Digital Content, http://links.lww.com/MD/O440).

## 4. Discussion

### 4.1. Effectiveness and acceptability

TCPMs exhibited favorable clinical outcomes in CAG treatment.^[72]^ However, there is still a lack of direct and indirect comparative research between these interventions. Therefore, we conducted a systematic review and network meta-analysis comparing 8 interventions for the treatment of CAG. This study evaluated the efficacy and safety of TCPMs combined with CT for CAG. A total of 49 studies were included, involving 5218 patients. The research results indicate that the combination of TCPMs + CT is effective. Additionally, it significantly improves clinical response rate and *H pylori* inhibition rate, while alleviating traditional Chinese medicine (TCM) symptoms in patients with CAG and reducing adverse reactions. Sensitivity analysis indicated that the results are relatively robust.

The methodology for this systematic review and network meta-analysis was rigorous and reproducible, with the literature search, eligibility judging, and data extraction performed independently by 2 investigators. Additionally, we used a random effects model to avoid overestimating the efficacy of treatments. In our primary analysis, there was no evidence of publication bias or other effects of small studies because the heterogeneity between studies was low or absent in almost all analyses.^[73]^

However, it could be argued that integrating these RCT outcomes into a meta-analysis may not be appropriate, given the diverse etiologies of CAG. Due to quantity and quality constraints, further research of superior quality is necessary to accumulate supplementary evidence. The standardization development of TCPMs and simple preparations also needs further improvement.

According to TCM theory, CAG is categorized under “epigastric pain”; it is believed to result from various factors such as food, emotions, and other factors that damage the spleen and stomach. This damage leads to biochemical powerlessness, deficiency of qi and blood, and loss of stomach nourishment. As a result, the stomach membrane is exposed to harmful chemicals, leading to prolonged stagnation of stomach collateral, finally contributing to CAG development.^[74]^ Notably, WFCT exhibited a significant improvement in symptoms by inhibiting gastric acid secretion and regulating pepsinogen levels.^[75–77]^

Additionally, *H pylori* infection was associated with atrophy, intestinal metaplasia, and GC. WFCT can mitigate mucosal inflammation, alleviate symptoms, and improve atrophy by regulating pro-inflammatory and anti-inflammatory factors.^[77]^ According to the study’s findings on network pharmacology and molecular coupling, WFCT may exhibit therapeutic potential in managing CAG through its multicomponent, multi-target, and multipathway mechanisms.^[78]^ Studies have also found that XSYWP can effectively inhibit gastric acid secretion and protect gastric mucosa.^[79]^ At the same time, XSYWP can reduce the serum inflammatory factor values, and inhibit inflammatory reactions, reducing GC incidence and alleviating its clinical symptoms.^[80]^

Unlike chemical drugs, TCM takes a holistic approach, offering a less toxic alternative with fewer side effects and enhanced efficacy in treating clinical symptoms. Compared with TCM decoctions, TCPMs are more convenient, and patients are more compliant with doctors when they prescribe TCPMs.^[81]^ Nevertheless, the composition of TCPMs remains constant, consequently rendering it impracticable to tailor them to specific individuals by including or excluding herbal elements. We hope to advance the development of more precise TCPMs, enabling personalized treatment strategies by leveraging enhanced symptom and disease classification systems.

### 4.2. Limitations

The current study was limited to the following: The included studies were most conducted and published in China, with some regional and linguistic bias; The dearth of comprehensive data regarding the registration of clinical trials rendered it unfeasible to ascertain the presence of selective reporting bias; The studies included in the review could have exhibited better quality, as most studies did not provide information on random sequence generation, and none reported allocation concealment and blinding, potentially undermining the dependability of their findings; The severity of the disease can cause heterogeneity in the studies, the treatment duration, and the chemical type used in CT; The lack of direct comparison of different TCPM and the wide 95% CI of effect size may affect the statistical effectiveness; In some studies, the follow-up time needed to be longer to evaluate the long-term efficacy of TCPM.

## 5. Conclusion

Our results demonstrate that, compared to using CT alone, TCPMs combined with CT can improve the clinical response rate in the treatment of CAG. Additionally, some TCPMs combined with CT can also enhance the *H pylori* inhibition rate. WST + CT and XSYWP + CT can even reduce the occurrence of adverse reactions associated with CAG. Using the CINeMA framework to evaluate the reliability of these endpoint outcomes revealed that most direct and indirect comparisons within the network were considered to have low or moderate levels of confidence. However, additional high-quality research is needed to obtain more accurate evidence due to the current limitations in both the quantity and quality of the included literature.

## Acknowledgments

I would like to express my gratitude to all those who helped me during the writing of this paper. A special Acknowledgments should be given to Professor Mengzhou Xie, from whose lectures I benefited greatly. I am particularly indebted to Professor Houpan Song, who provided me with kind encouragement and useful instruction throughout my writing.

## Author contributions

**Conceptualization:** Mengzhou Xie, Qinghua Peng.

**Data curation:** Baoping Ren, Meiqi Zhong, Chang Yu, Meng Xiong.

**Formal analysis:** Baoping Ren, Shunhua Zhou, Qing Gao, Xiaojuan Wang.

**Visualization:** Meiyan Zeng.

**Writing – original draft:** Baoping Ren.

**Writing – review & editing:** Houpan Song.

## Supplementary Material

## References

[1] Wei X Feng XP Wang LY. Improved method for inducing chronic atrophic gastritis in mice. World J Gastrointest Oncol. 2019;11:1115–25. 31908717 10.4251/wjgo.v11.i12.1115 PMC6937435

[2] Li J Pan J Xiao D. Chronic atrophic gastritis and risk of incident upper gastrointestinal cancers: a systematic review and meta-analysis. J Transl Med. 2024;22:429. 38711123 10.1186/s12967-023-04736-w PMC11075312

[3] Zheng SY Zhu L Wu LY. Helicobacter pylori-positive chronic atrophic gastritis and cellular senescence. Helicobacter. 2023;28:e12944. 36539375 10.1111/hel.12944

[4] Moayyedi P Lacy BE Andrews CN Enns RA Howden CW Vakil N. ACG and CAG clinical guideline: management of dyspepsia. Am J Gastroenterol. 2017;112:988–1013. 28631728 10.1038/ajg.2017.154

[5] Liu X Li D Wu J. Research progress in the treatment of chronic Atrophic gastritis with helicobacter pylori infection by traditional Chinese medicine in recent five years. Hebei J Tradit Chin Med. 2023;45:519–23.

[6] Wang B Zhou W Zhang H Wang W Zhang B Li S. Exploring the effect of Weifuchun capsule on the toll-like receptor pathway mediated HES6 and immune regulation against chronic atrophic gastritis. J Ethnopharmacol. 2022;303:115930. 36403744 10.1016/j.jep.2022.115930

[7] Wei W Yang Y Hospital W. Current situation of diagnosis & treatment for chronic atrophic gastritis and treating advantages of Chinese medicine. J Tradit Chin Med. 2016;57:36–40.

[8] Yin M Sui J Wang M. The efficacy and mechanism of action of Morodan concentrated pills in the treatment of chronic atrophic gastritis. J Mod Tradit Chin Med. 2024;44:98–109.

[9] Yang L Hu Z Zhu J Fei B. Effects of weifuchun tablet for chronic atrophic gastritis: A protocol for systematic review and meta-analysis. Medicine (Baltim). 2020;99:e20374. 10.1097/MD.0000000000020374 PMC12245332 32481421

[10] Wei X. Observation of the effect of Weisu granules on inflammatory response in patients with HP infected chronic gastritis. Mod Diagn Treat. 2024;35:639–41.

[11] Li T Su K Wang S. Modern research progress on Qizhi Weitong Granules. Chin J Experimental Pharm. 2024;30:145–52.

[12] Pan C. Analysis of the therapeutic effect of Xiangsha Yangwei Wan on patients with chronic atrophic gastritis. Chin Comm Doc. 2022;38:63–5.

[13] Witt CM. Clinical research on traditional drugs and food items--the potential of comparative effectiveness research for interdisciplinary research. J Ethnopharmacol. 2013;147:254–8. 23458921 10.1016/j.jep.2013.02.024

[14] Pedder H Dias S Bennetts M Boucher M Welton NJ. Joining the dots: linking disconnected networks of evidence using dose-response model-based network meta-analysis. Med Decis Making. 2021;41:194–208. 33448252 10.1177/0272989X20983315 PMC7879230

[15] Cipriani A Higgins JP Geddes JR Salanti G. Conceptual and technical challenges in network meta-analysis. Ann Intern Med. 2013;159:130–7. 23856683 10.7326/0003-4819-159-2-201307160-00008

[16] Page MJ McKenzie JE Bossuyt PM. The PRISMA 2020 statement: an updated guideline for reporting systematic reviews. BMJ. 2021;372:n71. 33782057 10.1136/bmj.n71 PMC8005924

[17] Hutton B Salanti G Caldwell DM. The PRISMA extension statement for reporting of systematic reviews incorporating network meta-analyses of health care interventions: checklist and explanations. Ann Intern Med. 2015;162:777–84. 26030634 10.7326/M14-2385

[18] Jansen JP Naci H. Is network meta-analysis as valid as standard pairwise meta-analysis? It all depends on the distribution of effect modifiers. BMC Med. 2013;11:159. 23826681 10.1186/1741-7015-11-159 PMC3707819

[19] Salanti G. Indirect and mixed-treatment comparison, network, or multiple-treatments meta-analysis: many names, many benefits, many concerns for the next generation evidence synthesis tool. Res Synth Methods. 2012;3:80–97. 26062083 10.1002/jrsm.1037

[20] Caldwell DM Ades AE Higgins JP. Simultaneous comparison of multiple treatments: combining direct and indirect evidence. BMJ. 2005;331:897–900. 16223826 10.1136/bmj.331.7521.897 PMC1255806

[21] Rucker G Schwarzer G. Ranking treatments in frequentist network meta-analysis works without resampling methods. BMC Med Res Methodol. 2015;15:58. 26227148 10.1186/s12874-015-0060-8 PMC4521472

[22] Higgins JP Jackson D Barrett JK Lu G Ades AE White IR. Consistency and inconsistency in network meta-analysis: concepts and models for multi-arm studies. Res Synth Methods. 2012;3:98–110. 26062084 10.1002/jrsm.1044 PMC4433772

[23] Chen W. Evaluation of the application of Lizhu stomach triple therapy combined with Weifuchun in the treatment of HP positive Atrophic gastritis. North Pharm. 2015;12:22–3.

[24] Lu D Chen Y Liu S Wang D. Effect of Weifuchun on helicobacter pylori positive chronic Atrophic gastritis and its influence on gastric juice pH and Pepsin. World Tradit Chin Med. 2018;13:2182–5.

[25] He F Liu F Zhang Y. Clinical analysis of gastric rejuvenation combined with triple anti Helicobacter pylori in the treatment of chronic Atrophic gastritis with peptic ulcer. Progress Mod Biomed. 2013;13:2352–5.

[26] Wang G. Clinical study on Weifuchun tablets combined with lansoprazole in treatment of chronic atrophic gastritis. Drugs Clin. 2019;34:1757–60.

[27] Wang H. Efficacy of Weifuchun tablet combined with quadri-combination therapy of rabeprazole in the treatment of chronic atrophic gastritis patients complicated with Hp infection. Acta Med Sinica. 2021;34:22–6.

[28] Li J Zhang D Zhao J. Clinical efficacy of Weifuchun tablet combined with lansoprazole on related serum indexes levels in the treatment of chronic atrophic gastritis. Med J West China. 2019;31:1043–7.

[29] Zhu J. Clinical evaluation of Moxabilli tablet and Weifuchun tablet in the treatment of chronic atrophic gastritis. Chin Foreign Med Res. 2019;17:29–31.

[30] Yang K. The clinical effect of Weifuchun tablets in the treatment of chronic atrophic gastritis. Inner Mongolia J Tradit Chin Med. 2020;39:46–7.

[31] Liu R Zhang L. Observation on efficacy of Weifuchun tablets combined with mosapride tablets in treatment of chronic atrophic gastritis. Eval Anal Drug-Use Hosp China. 2018;18:1195–7.

[32] Wang M. Clinical observation of the efficacy of lizhu stomach triple combination with gastric fuchun in the treatment of HP positive atrophic gastritis. Chin Mod Doc. 2007;45:46–56.

[33] Xu M Peng B Zhang C. Clinical study on the treatment of chronic atrophic gastritis with Weifuchun. Acta Chin Med. 2018;33:1537–41.

[34] Deng S. Effect of Weifuchun on chronic atrophic gastritis. J Clin Rat Drug Use. 2018;11:62–3.

[35] Wang S Wu F Tang Y. Clinical effect of Weifuchun combined with folic acid on chronic atrophic gastritis and its effect on serum pepsin and immune function. Mod Pract Med. 2020;32:349–50.

[36] Tang Y Pan Y Xue S. Clinical efficacy of Weifuchun combined with triple therapy in the treatment of patients with chronic atrophic gastritis and its effect on the serum gastrin, motilin and procalcitonin levels. Progress Mod Biomed. 2019;19:1903–6.

[37] Zang W. Effect of quadruple therapy combined with Weifuchun on serum osteopontin and gastrin in patients with chronic atrophic gastritis. Chin Med Herald. 2020;17:130–3.

[38] Huang X. Effect of quadruple therapy combined with gastric rejuvenation in the treatment of Hp Effect of quadruple therapy combined with gastric rejuvenation in the treatment of Hp positive chronic Atrophic gastritis. J Clin Med. 2017;4:10276–7.

[39] Long Y. Observation on the therapeutic effect of Weifuchun tablet on chronic Atrophic gastritis. J Med Theor Prac. 2019;32:998–9.

[40] Yifang W. Clinical efficacy of Weifuchun tablet combined with Lansoprazole in the treatment of chronic Atrophic gastritis and its effect on serum human soluble Interleukin 2 receptor. J Clin Rat Drug Use. 2020;13:55–6.

[41] Zhang S Zhu J Chen Q. Clinical study on the combination of Weifuchun tablets and compound proglumide cimetidine for chronic atrophic gastritis. Mod Drugs Clin Pract. 2019;34:1384–8.

[42] Jiwang Z Shiling D Weixing Z. Curative effect of folic acid tablet combined with Weifuchun tablet on precancerous lesions of chronic Atrophic gastritis. J New Chin Med. 2017;49:42–4.

[43] Geng L. Clinical observation on moluodan and vitamin b12 in the treatment of chronic atrophic gastritis and dysplasia. West Tradit Chin Med. 2012;25:11–3.

[44] QI L Zhou J Zhu L. Clinical study on Moluodan combined with itopride in treatment of chronic atrophic gastritis. Drugs Clin. 2022;37:1774–8.

[45] Shi Y Fu Q. Effect of folic acid combined with morodan on chronic atrophic gastritis. J North Pharm. 2017;14:91–2.

[46] Sun G Quan Y. MoLuoDan combined with vitamin B12 for the treatment of 56 cases of chronic atrophic gastritis. West J Tradit Chin Med. 2013;26:84–5.

[47] Wang W. Clinical efficacy of Moluo Dan and amoxicillin on chronic atrophic gastritis of Hp-positive. Clin J Chin Med. 2018;10:62–4.

[48] Wang L. Control effect of Morodan combined with Rabeprazole quadruple therapy on Hp positive chronic Atrophic gastritis. Chin J Public Health Eng. 2020;19:623–4.

[49] Zhang M Chen T Zhang L. Clinical observation on 30 cases of chronic atrophic gastritis treated with moloden and vitamin. J New Chin Med. 2016;48:44–6.

[50] Feng R. Clinical effect of Moluodan combined with folic acid on chronic atrophic gastritis with atypical hyperplasia. Hebei J Tradit Chin Med. 2011;33:865–7.

[51] Yue Y. Curative effect of morodan combined with anti helicobacter pylori drugs on chronic Atrophic gastritis. Inner Mongolia Med J. 2013;45:988–9.

[52] Xiao Z. Clinical observation on the treatment of chronic Atrophic gastritis with helicobacter pylori positive by morodan combined with quadruple therapy. Chin Naturopathy. 2019;27:67–8.

[53] Jiang C. Clinical value of Xiangsha Yangwei pill combined with teprenone capsules in the treatment of chronic atrophic gastritis. The Med Forum. 2018;22:3594–5.

[54] Wang H. Effect of Xiangsha Yangwei Pill on serum IL-8, IL-11 and TNF in patients with chronic Atrophic gastritis-α Horizontal impact. J North Pharm. 2017;14:33–4.

[55] Wu J Zhang M. Clinical study on Xiangsha Yangwei pills combined with lansoprazole for chronic atrophic gastritis of spleen deficiency and Qi stagnation type. J New Chin Med. 2020;52:39–42.

[56] Guo M Yang X. The clinical effect of Xiangsha Yangwei pill on chronic atrophic gastritis and its influence on serum inflammatory factors. Anti Infect Pharm. 2017;14:1080–2.

[57] Guo P Zhang Q. Effect of Xiangsha Yangwei Pill and Weifuchun tablet on inflammatory factors in patients with chronic atrophic gastritis. Med Laborat Clin. 2018;15:388–91.

[58] Song M Chen X. Clinical observation of Xiangsha Yangwei pills combined with lansoprazole in the treatment of spleen deficiency and Qi stagnation type chronic atrophic gastritis. Chin Pharm. 2017;36:5095–7.

[59] Kong X. Effect of Xiangsha Yangwei pill on chronic atrophic gastritis and inflammatory cytokines. Huaihai Med. 2018;36:726–8.

[60] He X Gong Y Hu Y. Clinical efficacy of Morodan combined with vitamin E in the treatment of chronic Atrophic gastritis with low-grade intraepithelial neoplasia. J Clin Digest Dis. 2017;29:294–6.

[61] Yang Y Wang B Ren Q. Clinical study on Xiangsha Yangwei pills combined with Teprenone capsules in treatment of chronic atrophic gastritis. Mod Med Clin Pract. 2017;32:71–5.

[62] Zhen H Yang S Luo G Shen B. Clinical observation on Xiangsha Yangwei pill combined with four combination therapy in treating HP positive chronic atrophic gastritis with gastric collateral blood stasis syndrome. Nei Mongol J Tradit Chin Med. 2022;41:11–2.

[63] Zhang M. Effect of Qizhi Weitong granule combined with Rabeprazole on chronic atrophic gastritis. Med J Chin People Health. 2021;33:68–9.

[64] Hu J. Effect of Qizhi Weitong granule and paroxetine hydrochloride on quality of life and prognosis recurrence rate of patients with chronic atrophic gastritis. J North Pharm. 2019;16:21–2.

[65] Zhao L. Observation on the effect of Qizhi Weitong granule combined with Teprenone in the treatment of chronic Atrophic gastritis. Henan Med Res. 2018;27:1099–100.

[66] Liu X Chen C Zhang Y. Clinical observation of Rabeprazole combined with Qizhi Weitong granules in the treatment of chronic atrophic gastritis patients. Chin J Clin Doc. 2020;48:156–9.

[67] Kan X Wang X. Rabeprazole combined with Qizhi Weitong Granules in the treatment of chronic Atrophic gastritis. J Chronic Dis. 2021;22:540–2.

[68] Lai Y Lan D. Clinical efficacy of Weisu granules in the treatment of chronic gastritis. J Clin Rat Drug Use. 2020;13:85–7.

[69] Pan Y Zhou H. Efficacy observation of Weisu particles in the treatment of chronic atrophic gastritis with spleen-stomach Qi block pattern. Clin Med Engin. 2015;22:885–6.

[70] Qi H. Effect of Weisu granules combined with quadruple therapy in the treatment of Hp positive chronic atrophic gastritis of the spleen and stomach with gas stagnation. Chin Mod Med. 2021;28:43–9.

[71] Tang C Lv X. Clinical study on the treatment of chronic atrophic gastritis with Weisu granules combined with vitamin E enzyme. Drugs Clin. 2019;34:2002–5.

[72] Chen Z Liu L. Research progress in the treatment of chronic atrophic gastritis with traditional Chinese medicine. Chin Naturopathy. 2022;30:117–9.

[73] Seitidis G Nikolakopoulos S Hennessy EA Tanner-Smith EE Mavridis D. Network meta-analysis techniques for synthesizing prevention science evidence. Prev Sci. 2022;23:415–24. 34387806 10.1007/s11121-021-01289-6

[74] Xu W Li D Du Y. Research on the etiology, pathogenesis, and pattern of syndrome elements of chronic atrophic gastritis based on the experience of renowned traditional Chinese medicine experts in contemporary Beijing Tianjin Hebei region. Liaoning J Tradit Chin Med. 2021;48:28–31.

[75] Zhang Y Ke Y Ye G. The clinical application and research status of Weifuchun tablets in the treatment of chronic atrophic gastritis and precancerous lesions of gastric cancer. Shanghai Pharm. 2018;39:43–6.

[76] Zhang S. Observation of the therapeutic effect of Weifuchun tablets combined with lansoprazole on patients with chronic atrophic gastritis. Mod Med Health Res. 2022;6:81–4.

[77] Gu Z Ling J Cong J Li D. A review of therapeutic effects and the pharmacological molecular mechanisms of Chinese medicine Weifuchun in treating precancerous gastric conditions. Integr Cancer Ther. 2020;19:1534735420953215. 32865036 10.1177/1534735420953215 PMC7466872

[78] Jiang X Lu T Luo Z. Mechanism of Weifuchun tablets in treating chronic atrophic gastritis based on network pharmacology and molecular docking and in vitro cell experiment verification. Drug Eval Res. 2022;45:1959–71.

[79] Ma Y. The effect of Banxia Xiexin Tang combined with Xiangsha Yangwei Wan in the treatment of chronic atrophic gastritis and its impact on serological indicators. Chin Health Engin. 2024;23:271–3.

[80] Wang H. The effect of Xiangsha Yangwei Wan on the levels of serum IL-8, IL-11, and TNF - α in patients with chronic atrophic gastritis. J Heze Med Coll. 2017;29:70–87.

[81] Yang L Gong J Chen W. Research progress on traditional Chinese medicine treatment of chronic atrophic gastritis. Clin J Chin Med. 2023;15:65–8.

