# Supplementary material for: Unveiling the therapeutic effects of traditional Chinese patent medicines: A network meta-analysis on chronic atrophic gastritis
Source: Medicine (Baltimore). 2025 Mar 7;104(10):e41690. doi: 10.1097/MD.0000000000041690 (PMC11902981; doi:10.1097/MD.0000000000041690)

Supplementary Table 1. traditional Chinese Patent Medicine (TCPM) formulations for chronic atrophic gastritis (CAG).

**Medicines**	**Ingredients**	**TCM effects**	
Moluodan	The flower of *Nelumbo nucifera* Gaertn, the rhizome of *Atractylodes macrocephala* Koidz., the root of *Scrophularia microdonta* Franch, the root of *Lindera aggregata* (sims) Kosterm, the tuber of *Alisma plantago-aquatica L.*, the tuber of *Ophiopogon Japonicus* (Thunb.) , the root of , the whole herb of , the root of *Cynanchum otophyllum Schneid,* the stem of *Dendrobium nobile* Lindl., the root of the root of *Aralia quinquefolia var. notoginseng* Burkill, the root of *Sanguisorba officinalis* L., the root of *Fumaria officinalis* L., the pollen of *Typha angustifolia* L.	Harmonize stomach and lower adverse qi, invigorate the spleen and eliminate distension, and activate blood circulation	RC<br>T
Weisu Tablet	The stem of *Perilla Frutescens* (L.) Britton, the root of *Cyperus rotundus* L., the skin of *Citrus × aurantium f. deliciosa* (Ten.) M.Hiroe, the ripe fruits of *Citrus medica* L., the ripe fruits of *Citrus × aurantium* L., the fruits of *Citrus × limon* (L.) Osbeck, the mature seeds of *Areca catechu* L.	Regulate qi and eliminate distension	
Weifuchun Tablets	The root of *Talinum paniculatum* (Jacq.), the stem and leaf of *Isodon amethystoides* (Benth.) H.Hara, the ripe fruits of *Citrus × aurantium* L.	Invigorate the spleen and qi, activate blood, and detoxify	
Qizhiweitong	The root of *Bupleurum falcatum* L., the root of	This intervention aims to relieve liver discomfort,	

Granule	*Fumaria officinalis* L., the ripe fruits of *Citrus × aurantium* L., the root of *Cyperus rotundus* L., the root of *Paeonia lactiflora* Pall, the root of *Glycyrrhiza uralensis* Fisch. ex DC.	enhance gastric function, facilitate the flow of vital energy, and alleviate physical discomfort
Xiangshayangwei Pill	The root of *Rosa indica* L., the ripe fruits of *Wurfbainia villosa* (Lour.) Škorničk. & A.D.Poulsen, the rhizome of *Atractylodes macrocephala* Koidz., the skin of *Citrus × aurantium f. deliciosa* (Ten.) M.Hiroe, the rhizome of *Pinellia ternata* (Thunb.) Makino, *Cyperus rotundus* L., the ripe fruits of *Wurfbainia compacta* (Sol. ex Maton) Škorničk. & A.D.Poulsen, the ripe fruits of *Citrus × aurantium* L., the skin of *Magnolia officinalis* Rehder & E.H.Wilson, the aboveground of *Pogostemon cablin* (Blanco) Benth., the root of *Glycyrrhiza uralensis* Fisch. ex DC.	The impact of moderate temperature on the gastrointestinal system

Supplementary Table 2. Characteristics of trials included in this review.

First author /Year	Male /Female	Sample size (treatment group/ control group	Treatment group				Control group				Outcome
			Interventions	Age (years)	Course of disease	Endpoint	Interventions	Age (years)	Course of disease	Endpoint	
**WFCT**											
Chen WJ (2015)	51/31	41/41	4 pieces/tid	17–65	2 d–10 y	3 m	Triple therapy	17–63	2 d–9 y	3 m	A, B, and C
**WFCT + CT**											
Zhang S (2019)	62/36	49/49	4 pieces/tid	33–79	9 m–15 y	4 m	Compound Proglumide and Cimetidine Tablets	35–77	1–16 y	4 m	A, C, and D
Zang WG (2020)	37/28	33/32	4 pieces/tid	19–80	5 m–30 y	1 y	Quadruple therapy	18–79	0.5–21 y	1 y	A, B, and C
Yang KY (2020)	61/65	63/63	4 pieces/tid	25–68	/	3 m	Triple therapy	24–67	/	3 m	A and B
Xu MX (2018)	151/89	120/120	4 pieces/tid	29–62	3 m–3 y	2 m	Triple therapy	30–64	3 m–3 y	2 m	A, B, and D
Wu YF (2020)	55/39	47/47	4 pieces/tid	60–79	3–12 y	4 w	Lansoprazole Enteric-coated Tablets	60–75	2–10 y	4 w	A and C
Deng SW (2018)	33/47	40/40	4 pieces/tid	40–67	/	2 w	Quadruple therapy	39–67	/	2 w	A and B
He FL (2013)	61/43	52/52	4 pieces/tid	31–72	/	4 w	Triple therapy	31–72	/	4 w	A, B, and C
Huang XY (2017)	109/89	99/99	4 pieces/tid	47.83 ± 4.39	7.72 ± 0.53 y	12 m	Quadruple therapy	47.30 ± 4.76	7.32 ± 0.45 y	12 m	A
Li JH (2019)	115/65	90/90	4 pieces/tid	30–58	1–14 y	2 w	Lansoprazole	28–55	1–12 y	2 w	A
Liu R (2018)	79/85	82/82	4 pieces/tid	46.7 ± 8.3	2.4 ± 1.8 y	4 m	Mosapride tablets	45.8 ± 7.9	2.5 ± 1.9 y	4 m	A and D

Long Y (2019)	51/45	48/48	4 pieces/tid	23–67	1–8 y	24 w	Triple therapy	22–66	1–9 y	24 w	A
Lu DW (2018)	119/81	81/86	1.44 g/tid	18–80	0.6–25 y	4 w	triple therapy	18–72	0.5–23 y	4 w	A
Tang YN (2019)	54/42	48/48	4 pieces/tid	26–68	1–14 y	4 w	Triple therapy	24–66	1–12 y	4 w	A
Wang MX (2007)	33/25	29/29	4 pieces/tid	/	/	3 m	Triple therapy	/	/	3 m	A, B, and C
Wang SY (2020)	29/31	30/30	4 pieces/tid	44–70	1–10 y	6 m	Folic acid tablets	40–69	2–11 y	6 m	A
Wang GF (2019)	40/22	31/31	4 pieces/tid	45–62	10 m–9 y	8 w	Lansoprazole Enteric-coated Tablets	42–58	8 m–11 y	8 w	A and D
Wang HX (2021)	53/45	49/49	4 pieces/tid	32–66	/	3 m	Quadruple therapy	31–65	/	3 m	A and C
Zhou JW (2017)	49/37	43/43	4 pieces/tid	24–65	1–7 y	24 w	Folic acid tablets	26–64	1–8 y	24 w	A
Zhu JJ (2019)	50/28	39/39	4 pieces/tid	30–66	2–13 y	14 d	Moxabilli Tablets	30–68	1–12 y	14 d	A and D
**MLD + CT**											
Li G (2012)	103/65	84/84	8 tablets/tid	27–63	5.6–21 y	3 m	Vitamin B12	26–69	4.7–25 y	3 m	A
Qi L (2022)	41/34	38/37	1 tablet/tid	35–72	0.6–12 y	3 m	Itopride Hydrochloride Dispersible Tablets	34–71	0.7–11 y	3 m	A and C
Shi YM (2017)	60/49	55/54	8 tablets/tid	39–69	1–12 y	3 m	Folic acid tablets	39–68	1–11 y	3 m	A
Sun GR (2013)	54/58	56/56	1 tablet/tid	36–68	1–5 y	3 m	Vitamin B12 + Vitacoenzyme Tablets	36–65	1–5 y	3 m	A and C
Wang FH (2018)	46/34	40/40	1 tablet/tid	40–72	3–15 y	2 w	Amoxicillin	40–72	3–15y	2 w	A and B
Wang L (2020)	98/86	92/92	1 tablet/tid	37–75	7 m–4 y	2 w	Quadruple therapy	36–77	9 m–4 y	2 w	A, B, and C
Xiao ZQ (2019)	49/43	46/46	1 tablet/tid	33–74	5 m–4 y	2 w	Quadruple therapy	35–72	6 m–5 y	2 w	A, B, and C
Zhang MM (2016)	29/31	30/30	8 tablets/tid	25–68	1–15 y	1 w	Vitacoenzyme Tablets	28–65	1–18 y	1 w	A and C

Yue YW (2013)		54/64	8 tablets/tid	/	4 y	12 w	Triple therapy	/	4.2 y	7 d	A and B
Feng RB (2011)	63/42	54/51	1 tablet/tid	27–63	3.8–22 y	3 m	Folic acid tablets	24–65	4.2–23 y	3 m	A and B
He XD (2017)	67/53	60/60	5 granule/tid	25–64	3.2–20 y	6 m	Vitamin E	27–66	4.1–21 y	6 m	A
**XSYWP**											
Wang HL (2017)	37/35	36/36	4 pieces/ tid	51.21 ± 8.34	8.42 ± 0.89 y	3 m	WFCT	50.91 ± 8.17	8.31 ± 0.92 y	3 m	A
Guo MG (2017)	53/37	45/45	4 pieces/tid	30–75	10 m–11 y	3 m	WFCT	29–76	9 m–11 y	3 m	A
Guo P. (2018)	50/42	46/46	8 tablets/tid	32–75	5 m–12 y	3 m	WFCT	30–77	6 m–13 y	3 m	A
Kong XC (2018)	51/39	45/45	8 tablets/tid	26–79	4–16 m	3 m	Teprenone	26–80	4–17 m	3 m	A and C
**XSYWP + CT**											
Song M (2017)	55/39	47/47	9 g/bid	60–79	3–12 y	4 w	Lansoprazole enteric-coated capsule	60–75	2–10 y	4 w	A and C
Jiang CM. (2018)	26/24	25/25	1 tablets/bid	41–70	6–15 y	30 d	Teprenone Capsules	42–72	6–16 y	30 d	A and C
Wu J (2020)	113/103	108/108	9 g/bid	49.37 ± 4.19	5.24 ± 1.67 y	4 w	lansoprazole	50.64 ± 4.25	5.30 ± 1.62 y	4 w	A and C
Yang Y (2017)	69/41	55/55	9 g/bid	48.6 ± 7.3	6 m–14 y	8 w	Teprenone Capsules	49.1 ± 7.0	8 m–13 y	8 w	A and C
Zhen HL (2022)	49/31	40/40	9 g/bid	64.22 ± 11.64	5.49 ± 1.15 y	2 w	Quadruple therapy	63.84 ± 10.23	5.28 ± 1.06 y	2 w	A, B, C, and D
**QZWTG + CT**											
Zhang MX (2021)	55/45	50/50	5 g/tid	31–67	1–10 y	3 m	Rabeprazole	30–67	1–9 y	3 m	A and C
Zhao LL (2018)	55/43	49/49	5 g/tid	25–67	8 m–7 y	13 d	Teprenone Capsules	26–68	9 m–8 y	13 d	A and B
Hu JB (2019)	53/41	47/47	5 g/tid	25–62	2–10 y	28 d	Paroxetine	24–61	1–9 y	28 d	A

							hydrochloride				
Kan X (2021)	55/41	48/48	5 g/tid	32–69	3–10 y	4 w	Rabeprazole	33–68	2–9 y	4 w	A and C
Liu XJ (2020)	38/30	34/34	5 g/tid	63.43 ± 6.79	4.08 ± 1.22 y	3 m	Rabeprazole	62.82 ± 7.01	3.99 ± 1.07 y	3 m	A and C
**WST**											
Lai YH (2020)	29/27	28/28	5 g/tid	31–76	7 m–5 y	1 m	Triple therapy	29–72	6 m–6 y	1 m	A and C
Pan YD (2015)	47/37	43/41	5 g/tid	25–64	/	4 w	Quadruple therapy	26–62	/	4 w	A and C
**WST + CT**											
Qi HL (2021)	75/41	58/58	5 g/tid	34–55	/	2 w	Quadruple therapy	35–60	/	2 w	A and C
Tang CL (2019)	63/79	71/71	5 g/tid	30–68	1–15 y	30 d	Vitacoenzyme Tablets	28–67	1–17 y	30 d	A

Note: A: clinical response rate; B: inhibition rate of *H. pylori;* C: adverse events; D: symptom scores. WFCT: Weifuchun Tablet; MLD: Moluodan; XSYWP: Xiangshayangwei Pill; QZWTG: Qizhiweitong Granule; WST: Weisu tablet; CT: conventional treatment of chemical drugs.

Supplementary Table 3. Confidence in network meta-analysis (CINeMA) framework evaluating the confidence in the indirect and direct treatment estimates from the network of randomized controlled trials (RCTs) of drugs in chronic atrophic gastritis (CAG).

Comparison	Number of Studies	Within-study bias	Reporting bias	Indirectness	Imprecision	Heterogeneity	Incoherence	Confidence rating	Reason(s) for downgrading
Mixed evidence									
CT vs. MLD + CT	11	Some concerns	Some concerns	No concerns	No concerns	No concerns	Major concerns	Moderate	Within-study bias and Reporting bias
CT vs. QZWTG + CT	5	Major concerns	Some concerns	Some concerns	Some concerns	No concerns	Major concerns	Low	Within-study bias, Reporting bias, and Indirectness
CT vs. WFCT	1	Some concerns	Some concerns	Major concerns	Major concerns	No concerns	Major concerns	Moderate	Incoherence
CT vs. WFCT + CT	19	Some concerns	Some concerns	No concerns	No concerns	No concerns	Major concerns	Moderate	Reporting bias
CT vs. WST	2	Major concerns	Some concerns	Some concerns	Some concerns	Some concerns	Major concerns	Low	Within-study bias and Imprecision
CT vs. WST + CT	2	Major concerns	Some concerns	Some concerns	Some concerns	Some concerns	Major concerns	Low	Within-study bias, Reporting bias, and Imprecision
CT vs. XSYWP	1	Some concerns	Some concerns	Some concerns	Some concerns	No concerns	No concerns	Moderate	Imprecision
CT vs. XSYWP + CT	5	Some concerns	Some concerns	No concerns	Some concerns	No concerns	Major concerns	Moderate	Imprecision
WFCT vs. XSYWP	3	Some concerns	Some concerns	Some concerns	Some concerns	Some concerns	Major concerns	Moderate	Incoherence
Indirect evidence									

MLD + CT vs. QZWTG + CT	--	Some concerns	Some concerns	Some concerns	Major concerns	Some concerns	Major concerns	Low	Imprecision and Incoherence
MLD + CT vs. WFCT	--	Some concerns	Some concerns	Some concerns	Major concerns	Some concerns	Major concerns	Low	Imprecision and Incoherence
MLD + CT vs. WFCT + CT	--	Some concerns	Some concerns	Some concerns	Major concerns	Some concerns	Major concerns	Low	Imprecision and Incoherence
MLD + CT vs. WST	--	Some concerns	Some concerns	Some concerns	Major concerns	Some concerns	Major concerns	Low	Imprecision and Incoherence
MLD + CT vs. WST + CT	--	Some concerns	Some concerns	Some concerns	Major concerns	Some concerns	Major concerns	Low	Imprecision and Incoherence
MLD + CT vs. XSYWP	--	Some concerns	Some concerns	Some concerns	Major concerns	Some concerns	Major concerns	Low	Imprecision and Incoherence
MLD + CT vs. XSYWP + CT	--	Some concerns	Some concerns	Some concerns	Major concerns	Some concerns	Major concerns	Low	Imprecision and Incoherence
QZWTG + CT vs. WFCT	--	Some concerns	Some concerns	Some concerns	Major concerns	Some concerns	Major concerns	Low	Imprecision and Incoherence
QZWTG + CT vs. WFCT + CT	--	Some concerns	Some concerns	Some concerns	Major concerns	Some concerns	Major concerns	Low	Imprecision and Incoherence
QZWTG + CT vs. WST	--	Some concerns	Some concerns	Some concerns	Major concerns	Some concerns	Major concerns	Low	Imprecision and Incoherence
QZWTG + CT vs. WST + CT	--	Some concerns	Some concerns	Some concerns	Major concerns	Some concerns	Major concerns	Low	Imprecision and Incoherence
QZWTG + CT vs. XSYWP	--	Some concerns	Some concerns	Some concerns	Major concerns	Some concerns	Major concerns	Low	Imprecision and Incoherence
QZWTG + CT vs. XSYWP + CT	--	Some concerns	Some concerns	Some concerns	Major concerns	Some concerns	Major concerns	Low	Imprecision and Incoherence

WFCT vs. WFCT + CT	--	Some concerns	Some concerns	Some concerns	Major concerns	Some concerns	Major concerns	Low	Imprecision and Incoherence
WFCT vs. WST	--	Some concerns	Some concerns	Some concerns	Major concerns	Some concerns	Major concerns	Low	Imprecision and Incoherence
WFCT vs. WST + CT	--	Some concerns	Some concerns	Some concerns	Major concerns	Some concerns	Major concerns	Low	Imprecision and Incoherence
WFCT vs. XSYWP + CT	--	Some concerns	Some concerns	Some concerns	Major concerns	Some concerns	Major concerns	Low	Imprecision and Incoherence
WFCT + CT vs. WST	--	Some concerns	Some concerns	Some concerns	Major concerns	Some concerns	Major concerns	Low	Imprecision and Incoherence
WFCT + CT vs. WST + CT	--	Some concerns	Some concerns	Some concerns	Major concerns	Some concerns	Major concerns	Low	Imprecision and Incoherence
WFCT + CT vs. XSYWP	--	Some concerns	Some concerns	Some concerns	Major concerns	Some concerns	Major concerns	Low	Imprecision and Incoherence
WFCT + CT vs. XSYWP + CT	--	Some concerns	Some concerns	Some concerns	Major concerns	Some concerns	Major concerns	Low	Imprecision and Incoherence
WST vs. WST + CT	--	Some concerns	Some concerns	Some concerns	Major concerns	Some concerns	Major concerns	Low	Imprecision and Incoherence
WST vs. XSYWP	--	Some concerns	Some concerns	Some concerns	Major concerns	Some concerns	Major concerns	Low	Imprecision and Incoherence
WST vs. XSYWP + CT	--	Some concerns	Some concerns	Some concerns	Major concerns	Some concerns	Major concerns	Low	Imprecision and Incoherence
WST + CT vs. XSYWP	--	Some concerns	Some concerns	Some concerns	Major concerns	Some concerns	Major concerns	Low	Imprecision and Incoherence
WST + CT vs. XSYWP + CT	--	Some concerns	Some concerns	Some concerns	Major concerns	Some concerns	Major concerns	Low	Imprecision and Incoherence

XSYWP vs. XSYWP + CT	--	Some concerns	Some concerns	Some concerns	Major concerns	Some concerns	Major concerns	Low	Imprecision and Incoherence

Note: WFCT: Weifuchun Tablet; MLD: Moluodan; XSYWP: Xiangshayangwei Pill; QZWTG: Qizhiweitong Granule; WST: Weisu tablet; CT: conventional treatment of chemical drugs.

Supplemental Figure 1. Flow chart of the statistical analysis.

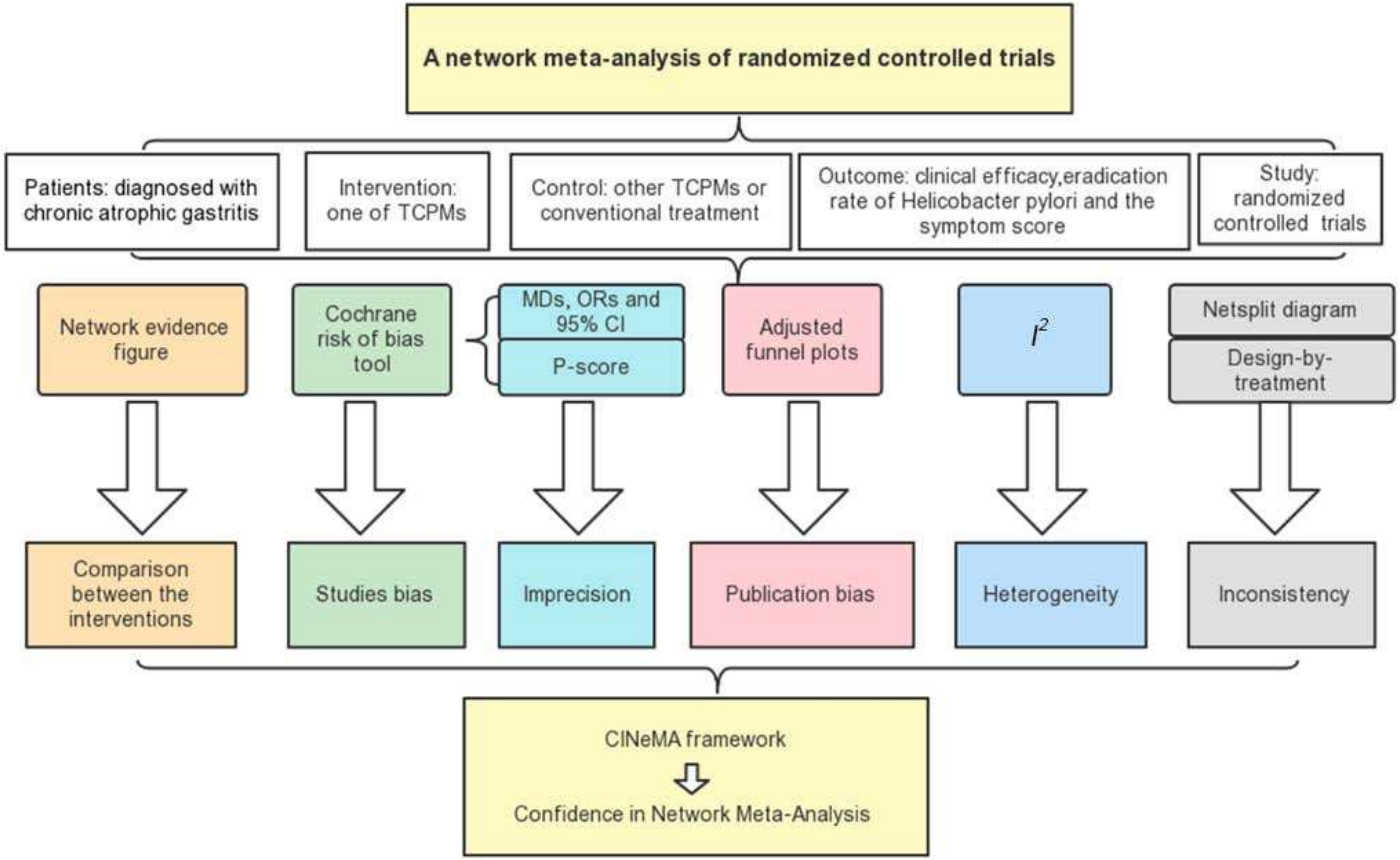

Supplemental Figure 2. Risk of bias. green represents low risk, yellow represents unclear risk, and red represents high risk.

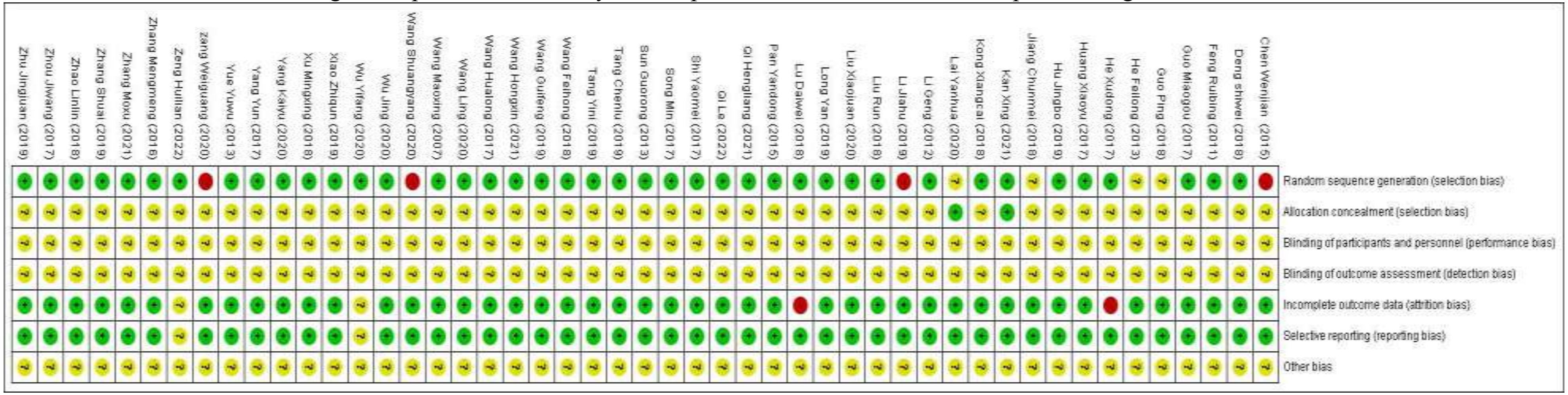

Supplemental Figure 3. Forest plot for pairwise comparison of **(A)** clinical response rate, **(B)** the inhibition rate of *H. pylori*, and **(C)** adverse events. The line segment marked green indicates a statistically significant difference between the two intervention measures.

(A and C). A: conventional treatment (CT) of chemical drugs; B: Moluodan + CT; C: Qizhiweitong Granule + CT; D: Weifuchun Tablet; E: Weifuchun Tablet + CT; F: Weisu tablet; G: Weisu Tablet + CT; H: Xiangshayangwei Pill; I: Xiangshayangwei Pill + CT.

(B). A: conventional treatment of chemical drugs; B: Moluodan + CT; C: Qizhiweitong Granule + CT; D: Weifuchun Tablet Tablet; E: Weifuchun Tablet + CT; F: Xiangshayangwei Pill + CT.

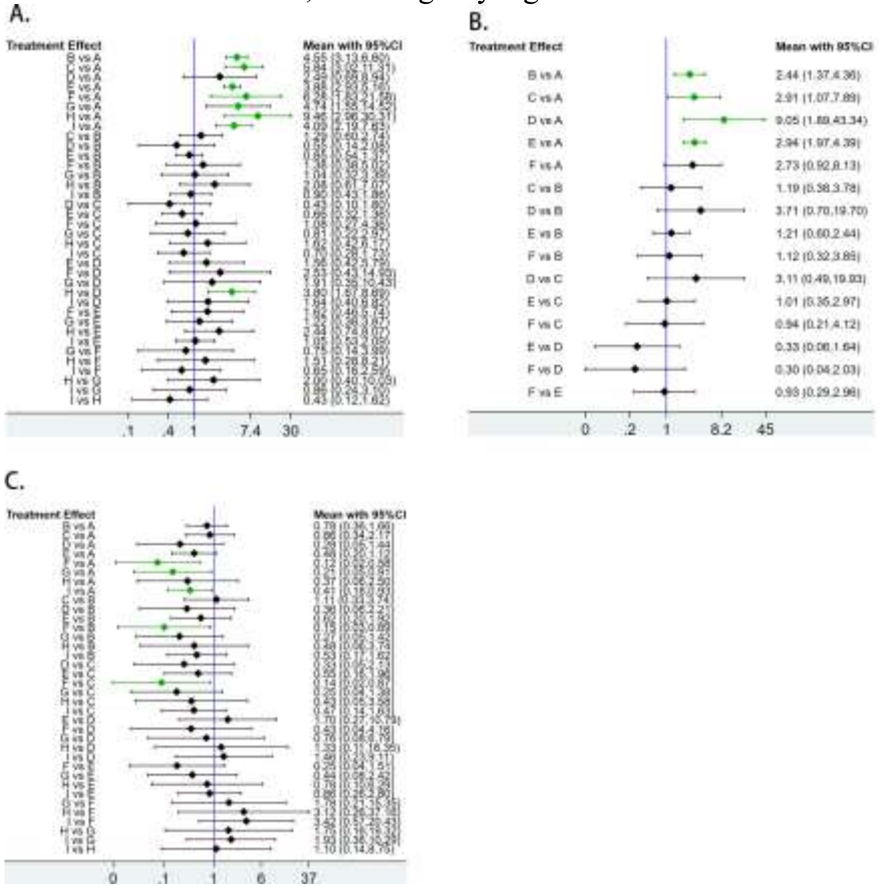

Supplemental Figure 4. Forest plot of a symptom score for **(A)** Bitter taste and dry throat, **(B)** Gastric noise, and **(C)** Belch and acid reflux.

A

Study or Subgroup	Experimental Mean	Experimental SD	Experimental Total	Control Mean	Control SD	Control Total	Weight	Mean Difference IV, Random, 95% CI
Liu Run(2018)	-1.1	0.05	82	-0.07	0.02	80	25.3%	-1.03 [-1.04, -1.02]
Xu Mingxing(2018)	-1.92	0.3	120	-1.58	0.26	120	24.6%	-0.34 [-0.41, -0.27]
Zhang Shuai(2019)	-1.82	0.14	49	-1.07	0.09	49	25.0%	-0.75 [-0.80, -0.70]
Zhu Jingjuan(2019)	-1.08	0.06	39	-0.12	0.08	39	25.2%	-0.96 [-0.99, -0.93]
**Total (95% CI)**			**290**			**288**	**100.0%**	**-0.77 [-0.98, -0.57]**

Heterogeneity: Tau^2 = 0.04; Chi^2 = 471.95, df = 3 (P < 0.00001); I^2 = 99%
Test for overall effect: Z = 7.48 (P < 0.00001)

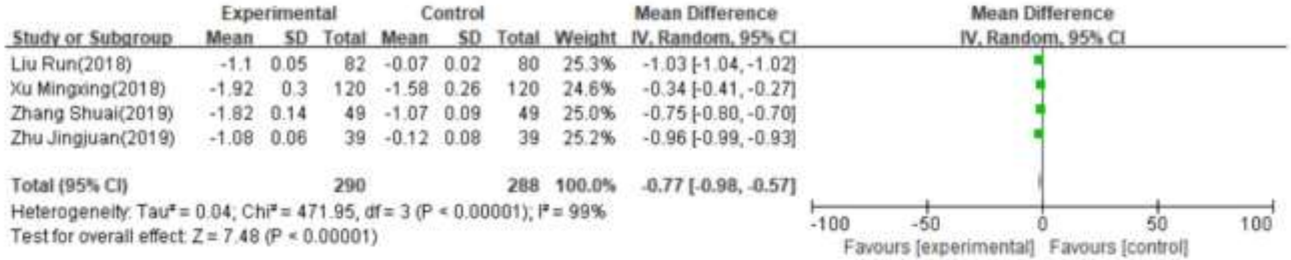

B

Study or Subgroup	Experimental Mean	Experimental SD	Experimental Total	Control Mean	Control SD	Control Total	Weight	Std. Mean Difference IV, Random, 95% CI
Liu Run(2018)	-1.55	0.16	82	-0.92	0.1	80	69.3%	-4.69 [-5.29, -4.09]
Zhu Jingjuan(2019)	-1.5	0.1	39	-0.9	0.14	39	30.7%	-4.88 [-5.79, -3.98]
**Total (95% CI)**			**121**			**119**	**100.0%**	**-4.75 [-5.25, -4.25]**

Heterogeneity: Tau^2 = 0.00; Chi^2 = 0.13, df = 1 (P = 0.72); I^2 = 0%
Test for overall effect: Z = 18.59 (P < 0.00001)

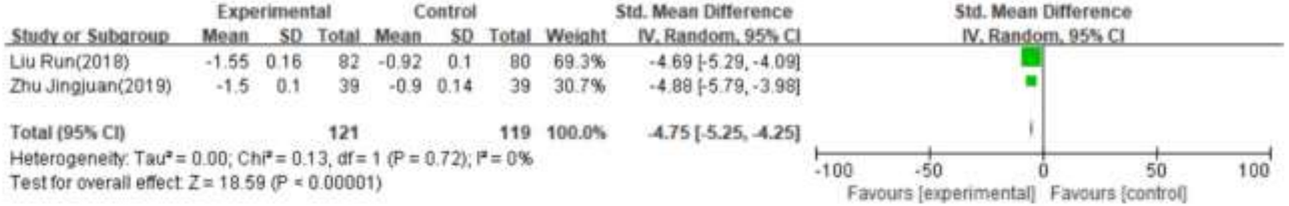

C

Study or Subgroup	Experimental Mean	Experimental SD	Experimental Total	Control Mean	Control SD	Control Total	Weight	Std. Mean Difference IV, Random, 95% CI
Liu Run(2018)	-1.94	0.12	82	-1.06	0.07	80	25.1%	-8.89 [-9.92, -7.86]
Xu Mingxing(2018)	-2.18	0.25	120	-1.91	0.26	120	25.4%	-1.06 [-1.33, -0.78]
Zhang Shuai(2019)	-1.26	0.07	49	-0.62	0.16	49	25.2%	-5.14 [-5.98, -4.31]
Zhu Jingjuan(2019)	-1.9	0.02	39	-1.08	0.1	39	24.3%	-11.26 [-13.13, -9.39]
**Total (95% CI)**			**290**			**288**	**100.0%**	**-6.53 [-10.97, -2.09]**

Heterogeneity: Tau^2 = 20.17; Chi^2 = 368.51, df = 3 (P < 0.00001); I^2 = 99%
Test for overall effect: Z = 2.88 (P = 0.004)

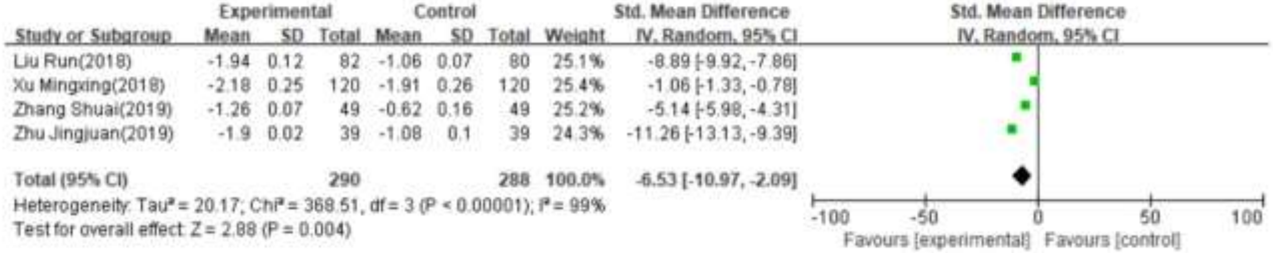

Supplemental Figure 5. (A) Network plot for relieving pain score for the stomach pain in chronic atrophic gastritis (CAG): all randomized controlled trials (RCTs). The circle (node) size is proportional to the number of study participants assigned to receive each intervention. The line width (connection size) corresponds to the number of studies comparing the individual interventions. **(B)** Forest plot for relieving pain score for the stomach pain in chronic atrophic gastritis (CAG): all randomized controlled trials (RCTs).
WFCT: Weifuchun Tablet; XSYWPCT: Xiangshayangwei Pill; CT: conventional treatment of chemical drugs.

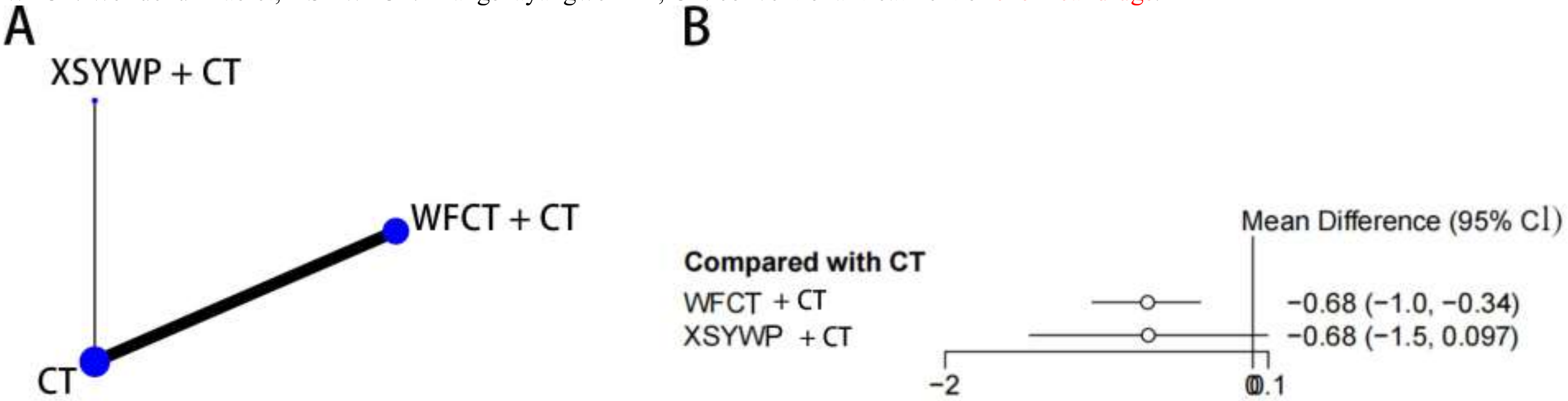

Supplemental Figure 6. Forest plot for adverse events in chronic atrophic gastritis (CAG): all randomized controlled trials (RCTs). The P-score is the probability of each intervention being ranked best in the network.

WFCT: Weifuchun Tablet; MLD : Moluodan; XSYWP: Xiangshayangwei Pill; QZWTG: Qizhiweitong Granule; WST: Weisu tablet; CT: conventional treatment of chemical drugs.

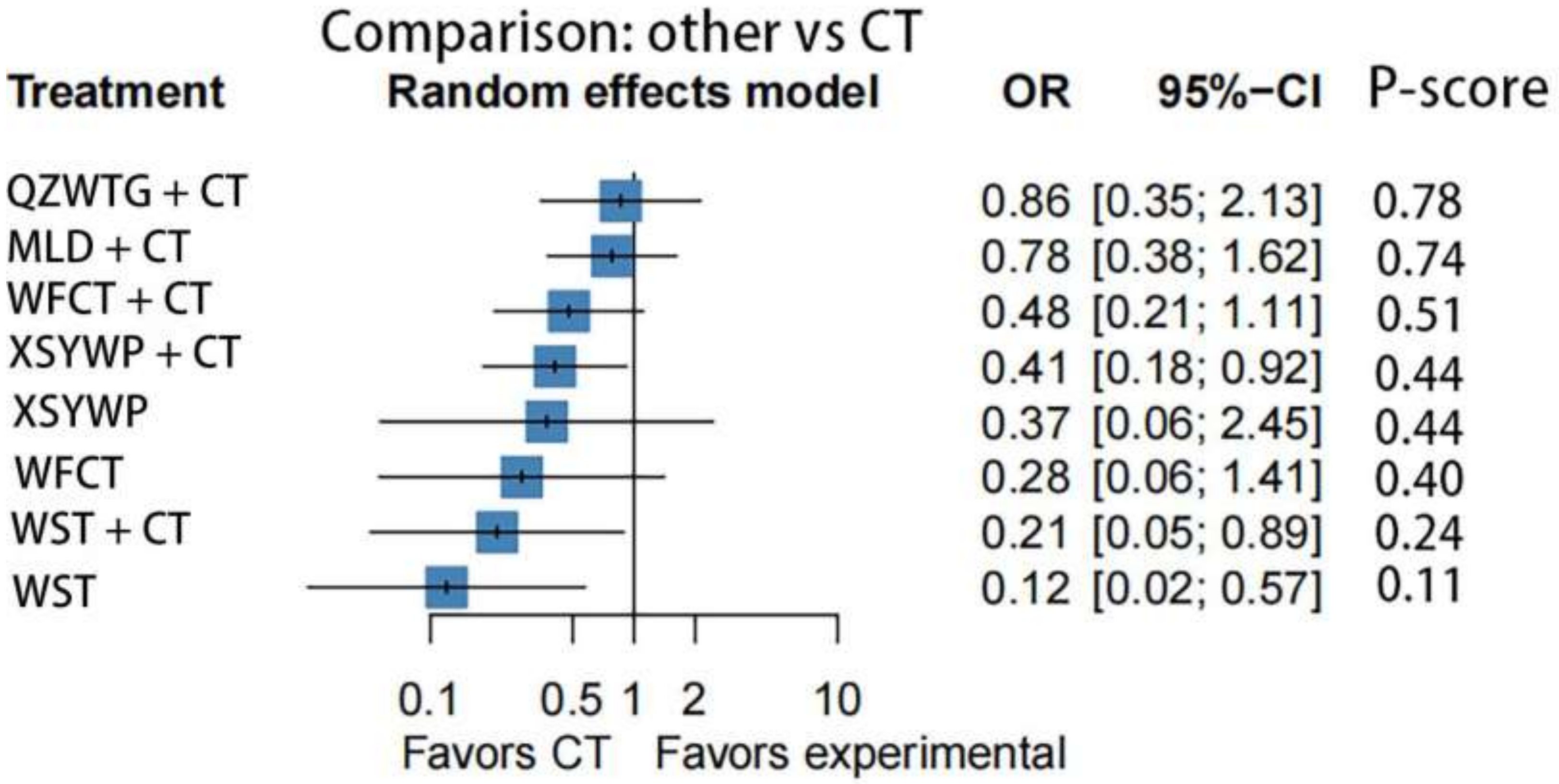

Supplemental Figure 7. Sensitivity analysis chart of clinical response rate.

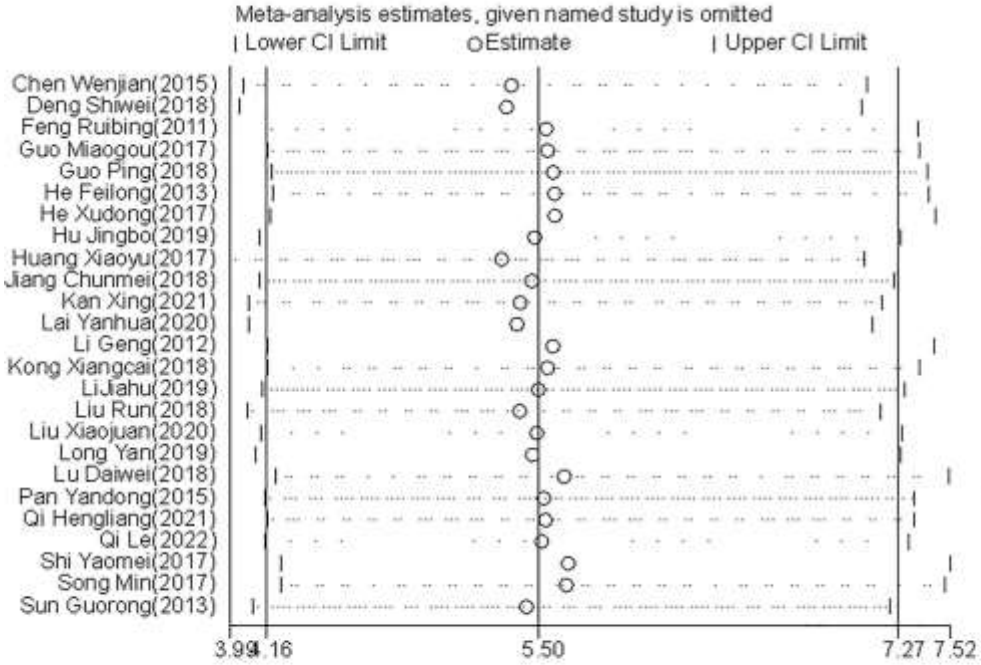

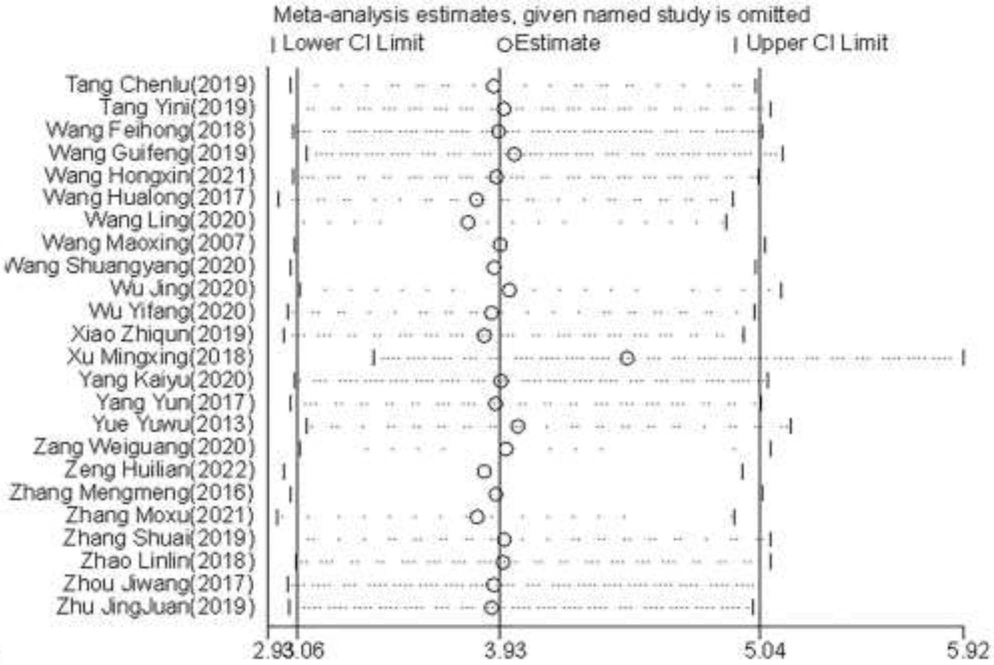

Supplement: Supplementary file 1 [file medi-104-e41690-s001.pdf]
